# Thyroid Storm with Multiorgan Failure Treated with Plasmapheresis

**DOI:** 10.1155/2019/2475843

**Published:** 2019-10-09

**Authors:** Ann Miller, Kristi D. Silver

**Affiliations:** Division of Endocrinology, Diabetes, and Nutrition, Department of Medicine, University of Maryland School of Medicine, Baltimore, MD, USA

## Abstract

**Background:**

Thyroid storm is a severe manifestation of thyrotoxicosis and can present with multiorgan failure. First line treatment of thyroid storm is directed towards decreasing thyroid hormone production and peripheral conversion of T4 to T3, and treating adrenergic symptoms. When medical therapy fails, plasmapheresis is an alternative treatment option. Here we present a patient with thyroid storm and multiorgan failure who was treated with plasmapheresis.

**Case:**

A 50-year-old male with a history of hyperthyroidism, hypertension, and congestive heart failure presented to another hospital with fever and altered mentation. He was found to have pneumonia on imaging and was started on antibiotics. He developed shock complicated by atrial fibrillation with rapid ventricular rate which was treated with amiodarone. He was transferred to our hospital for further management. On arrival, TSH was <0.01 mIU/L, free T4 was >7 ng/dL and total T3 was 358 ng/dL. The endocrinology team determined he was in thyroid storm. His medical treatment of thyroid storm was aggressively titrated to maximal therapy. His hospital course was complicated by transaminitis, respiratory failure requiring intubation, shock requiring vasopressor support, kidney failure requiring continuous renal replacement therapy, and heart failure. Despite maximal anti-thyroid therapy, he had not improved clinically and T4 and T3 remained markedly elevated. A 4-day course of plasmapheresis was initiated resulting in marked lowering of T4 and T3 and clinical stability.

**Conclusion:**

While current guidelines for plasmapheresis for thyroid storm recommend individualized decision making, no further clarification is provided on who would be a good candidate for the procedure. We present a patient with thyroid storm and multiorgan failure who was treated with plasmapheresis after failing maximal medical therapy. Given the significant improvement seen with plasmapheresis, endocrinologists should consider this mode of treatment earlier in the course of thyroid storm when patients are not improving with medical therapy alone.

## 1. Background

Thyroid storm is a severe manifestation of thyrotoxicosis and can present with multiorgan failure. Thyroid storm has an estimated mortality rate of 20%–30% [1]. First line treatment of thyroid storm is directed at decreasing thyroid hormone production and peripheral conversion of thyroxine (T4) to triiodothyronine (T3), and treating adrenergic symptoms. When medical therapy fails, therapeutic plasma exchange (TPE), also called therapeutic plasmapheresis, is an alternative treatment option. Here we present a patient with thyroid storm and multiorgan failure who was successfully treated with TPE.

## 2. Case

A 50-year-old African American male with a history of hyperthyroidism, hypertension, and congestive heart failure presented to an outside hospital with fever and an altered mental status. He was diagnosed with hyperthyroidism about three months prior to hospitalization. He was started on methimazole (MMI), but compliance taking the medication was low. His primary care provider had recommended thyroidectomy; however, he was unable to have the procedure due to lack of health insurance. On presentation to the outside hospital, imaging revealed right lower lobe pneumonia with an effusion and he was started on antibiotics. His clinical status deteriorated, and he developed shock complicated by atrial fibrillation with rapid ventricular rate with documented rates in the 140–190 beats per minute. His arrhythmia was refractory to digoxin, diltiazem, and two attempts at cardioversion with 200 Joules. He was initiated on an amiodarone infusion which stabilized his arrhythmia. His TSH documented at the outside hospital was 0.01 mIU/L and free T4 was 8 ng/dL.

He was transferred to our hospital for further management. Prior to transfer, he was started on hydrocortisone 50 mg every 6 hours and MMI 10 mg three times daily. MMI was used instead of propylthiouracil (PTU) due to elevated liver function tests. On the day of arrival to our hospital, the inpatient endocrinology team was consulted for assistance with thyroid management. He was intubated for respiratory distress at the time of the endocrinology team's initial assessment. His blood pressure, supported by two pressors, was 90/63 mmHg. His temperature was 36.9˚C and his pulse ranged from 88 to 134 beats per minute on the amiodarone infusion. Physical examination was significant for scleral icterus and left neck fullness. No thyroid bruit or discrete nodules were identified; however, the neck exam was limited due to multiple central lines. His heart beat was irregular consistent with atrial fibrillation and a cardiac murmur was also detected. Lower extremities were notable for edema and hyperreflexia. The endocrinology team was unable to assess his mental status due to the patient being sedated. Thyroid labs on admission to our hospital included TSH <0.01 mIU/L (0.47–4.68 mIU/L), total T3 358 ng/dL (97–169 ng/dL), free T4 > 7 ng/dL (0.6–2.5 ng/dL) and thyroid stimulating antibodies >500% (normal ≤122%). Additional laboratory studies (Table 1) showed acute kidney injury and elevated liver function tests, troponin, and white blood cell count. Thyroid ultrasound with doppler showed an enlarged, heterogeneous thyroid gland, more pronounced on the right than the left without any nodules, although the view of the left side was limited due to the endotracheal tube and central line.

His Burch–Wartofsky score [2] for hyperthyroid storm was 100 which was highly concerning for thyroid storm. As the Burch–Wartofsky score can sometimes be misleading with elevated scores in critically ill patients without thyroidal illness, the Japan Thyroid Association thyroid storm criteria, a diagnostic criterion less likely to incorrectly diagnose thyroid storm in critically ill patients, was also utilized to assess for thyroid storm in our patient [3]. Employing this second diagnostic criterion, our patient again met criteria for thyroid storm based on the family report of altered mental status, tachycardia with documented rates of 140–190 per minute, heart failure with an estimated ejection fraction of 30%, and thyrotoxicosis with suppressed TSH and elevated free T4 and total T3 levels.

The endocrine consult team recommended increasing MMI to 20 mg three times daily and starting cholestyramine 4 g three times daily. Over the ensuing days, his renal function worsened such that he required continuous renal replacement therapy. On day 2, saturated potassium iodide solution (SSKI) 250 mg three times daily was initiated and cholestyramine was increased to 4 g four times daily. Liver function tests improved by day 3; therefore, he was switched from MMI to PTU 200 mg three times daily to take advantage of the decreased conversion of T4 to T3 seen with PTU. On day 4, TSH remained undetectable and free T4 was unchanged at >7 ng/dL. Total T3 began to decrease, but remained >200 ng/dL. Despite maximal medical therapy for thyroid storm, his clinical status continued to worsen with evidence of multiorgan failure. The endocrinology team consulted the hematology physicians for initiation of TPE.

**Figure 1:**
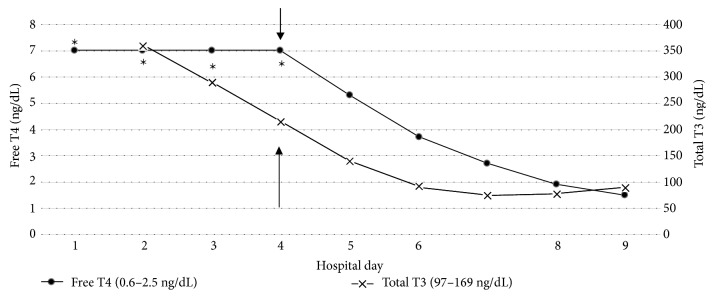
Free T4 and total T3 Levels during hospitalization. ^∗^Values were >7 ng/dL, → first day of plasmapheresis.

He was started on daily TPE on hospital day 4. Replacement fluid contained half fresh frozen plasma (FFP) and half 5% albumin and was equal in volume to his total plasma volume (about 4L). His TPE treatment lasted about two hours each day and he received plasmapheresis daily for four days. After the first day of TPE, his free T4 and total T3 decreased to 5.3 ng/dL and 139 ng/dL, respectively. After 4 sessions of daily TPE, his free T4 was 1.9 ng/dL and total T3 was 77 ng/dL (Figure 1). With TPE, his clinical course significantly improved to the point where he was weaned off of pressors and extubated. Over the following 2 weeks, he was safely titrated off of SSKI and hydrocortisone, and switched from PTU to MMI. For definitive treatment of hyperthyroidism, the endocrine surgery team was consulted to evaluate the patient for total thyroidectomy. At the time of their evaluation, the patient was clinically euthyroid. Given his recent critical illness, the surgeons considered him to be a high-risk surgical candidate. Therefore, they recommended performing an elective thyroidectomy once the patient was further out from his critical illness and had undergone outpatient cardiac optimization. He was discharged home three weeks later on MMI 30 mg twice daily and cholestyramine 4 g twice daily. At time of discharge, he did not require any supplemental oxygen and his left ventricular ejection fraction improved to 48% as measured on positron emission tomography myocardial perfusion scan. He was no longer in atrial fibrillation as he had converted back to sinus rhythm and amiodarone had been discontinued. With the exception of total bilirubin, his liver function tests had normalized before discharge. By 2 months post discharge, his total bilirubin had also normalized. He was discharged with intermittent dialysis needs, though two weeks after leaving the hospital, dialysis was discontinued because his kidney function recovered.

## 3. Discussion

Plasmapheresis is an extracorporeal blood purification technique that helps remove larger molecular weight substances from blood. Plasmapheresis is a general term that refers to removing plasma from blood. TPE is a type of plasmapheresis that involves removal of patient plasma and replacing it with something else (either donor plasma, colloid, or crystalloid). TPE is most often used to treat conditions, where a pathogenic substance or component is in the blood and needs to be rapidly removed. Examples of pathogenic substances include auto-antibodies, immunocomplexes, cryoglobulins, myeloma light chains, endotoxins, cholesterol containing lipoproteins, and as with our patient, plasma protein-bound thyroid hormones. The necessary tools for TPE include vascular access, either using large-bore needles in the limb veins or an implanted catheter in the large veins of the neck, chest, or groin, and a plasmapheresis machine. The machine separates the patient's plasma from the rest of the blood components and exchanges plasma with a replacement fluid. TPE is effective for treatment of thyroid storm as thyroid hormone is almost entirely bound to plasma proteins (99.97% of total serum T4 and 99.7% of total serum T3). The three main plasma proteins that bind T4 and T3 are thyroxine binding globulin (TBG), transthyretin, and albumin. TBG binds 75% of T4 and T3. Transthyretin binds 20% of T4 and <5% of T3. Albumin binds 5% of T4 and 20% of T3. TBG and transthyretin are relatively similar in size, 54 kDa and 55 kDa, respectively. Albumin is larger with a molecular mass of 66.5 kDa [4]. Because of the size of thyroid binding proteins, conventional dialysis cannot remove these proteins from blood. In contrast, TPE removes larger proteins, and thus can be used to reduce circulating thyroid hormone and treat refractory thyroid storm. TPE can also remove TSH receptor autoantibody (also referred to as thyroid stimulating immunoglobulin) and its removal has been predicted mathematically to result in rapid lowering of the free thyroxine levels [5].

Randomized placebo-controlled studies of TPE with medical management resistant thyroid storm have not been performed due to the rarity of the disorder. Therefore, guidelines for the use of TPE for thyroid storm are based on case reports, retrospective studies, and expert opinion. According to The American Society for Apheresis 2016 clinical practice guidelines, treatment of thyroid storm with plasmapheresis is a category III indication [6] meaning that the optimal role of TPE in thyroid storm is not established and the decision to use TPE for this indication should be individualized. Per these guidelines, when plasmapheresis is used to treat thyroid storm, FFP or albumin should be used as the replacement fluid. FFP has the benefit of increasing the concentration of TBG, which binds specifically to thyroxine and triiodothyronine, while albumin provides greater capacity for binding thyroid hormone. The recommended volume of replacement fluid is 1–1.5 times the total plasma volume. TPE treatment should be daily to every 3 days until clinical improvement is noted. TPE should be reserved for patients with severe symptoms of thyroid storm in whom first line medical therapies have failed or have had detrimental side effects that outweigh the benefits of treatment. These guidelines are similar to the guidelines set forth by The Japan Thyroid Association and Japan Endocrine Society in 2016 [7]. The Japanese guidelines recommend the use of TPE for treatment of thyroid storm if there is acute liver failure or no clinical improvement after 24–48 hours on optimized medical management. The Japanese guidelines recommend FFP over albumin as the replacement fluid due to its higher levels of TBG and specificity to bind thyroid hormone.

The effectiveness of TPE is dependent on multiple variables and is not a procedure without risk. The successful removal of pathogenic substances from blood through plasmapheresis depends on the volume of blood processed and plasma exchanged in each procedure, number of procedures performed, frequency of exchange, and rate of mobilization, stabilization, and re-synthesis of the cells or plasma components. Complications of plasmapheresis include bleeding, infection, disseminated intravascular coagulation (DIC), coagulation factor depletion leading to hypocoagulability, hypocalcemia, hypotension, and transfusion reactions such as transfusion associated infections, pulmonary edema, and pulmonary embolism. Despite these potential complications, the mortality rate of plasmapheresis is <1% [8].

Ashkar et al. first described thyrotoxicosis treated with plasmapheresis in 1970 in a case report of three thyrotoxic patients [9]. All three patients had deteriorating clinical status despite maximal medical treatment of thyrotoxicosis. The first patient underwent true TPE as his plasma was replaced with his red blood cells and lactate ringers. The other two patients were treated with plasmapheresis with removal of plasma with only their red blood cells returned to them. All three patients had significant clinical improvement. Since this initial description, case reports have described successful treatment with TPE in a variety of hyperthyroid scenarios ranging from post-operative thyroid storm complicated by pneumonia and septic shock [10] to MMI induced agranulocytosis [11] to amiodarone induced thyrotoxicosis [12]. While patients previously described as receiving plasmapheresis for thyroid storm were often critically ill, few have had multisystem organ failure similar to our patient. Of the few with multiorgan failure, one with a history of Graves' and medication noncompliance developed symptomatic thyrotoxicosis including palpitations, nausea, and vomiting. He was treated with medical therapy, but had to be switched from PTU to MMI due to liver dysfunction. He clinically decompensated despite medical therapy and developed DIC. He was treated with plasmapheresis and after two treatments, his free T4 and free T3 normalized. The same paper discusses a male with Graves' disease who also presented with thyrotoxicosis symptoms and medication noncompliance. This patient was started on oral medical therapy with MMI 20 mg daily and propranolol 40 mg twice daily. Shortly after admission, he became febrile with a temperature of 38.1°C and was found unresponsive and pulseless. He underwent cardiopulmonary resuscitation. He developed ventricular fibrillation and required electrical cardioversion. His clinical status further worsened with development of transaminitis and acute kidney injury with continued tachyarrhythmia. Neurologically, he was unresponsive off of sedation. After one day of plasmapheresis, his free T4 declined from 10.9 ng/dL to 8.57 ng/dL. He received two more treatments of plasmapheresis daily over the next two days. His free T4 continued to trend downwards as he continued to receive methimazole, SSKI, and steroids. While the paper does not talk about his neurological status after TPE, it did say that he became clinically and biochemically euthyroid and underwent definitive treatment with a total thyroidectomy [13]. Similarly, in a case reported by Baena et al., a 36-year-old woman with Graves' disease and multiorgan failure including acute liver and heart failure, acute kidney injury, cytopenia, rhabdomyolysis and coagulopathy improved with the use of medical management plus plasmapheresis [14].

Because of the infrequent use of TPE for treatment of thyroid storm/thyrotoxicosis, randomized controlled studies on its efficacy versus continuing with only maximal medical therapy have not yet been performed. Few retrospective reviews on TPE with thyroid storm involving more than one or two patients have been published. In a review by Ezer et al., 11 thyrotoxicosis patients successfully responded to pre-operative plasmapheresis treatment [15]. The etiologies of thyrotoxicosis (7-Graves' Disease, 3-toxic multinodular goiters, 1-iodine induced thyrotoxicosis) and the indications for plasmapheresis varied (7-poor response to medical therapy, 2-agranulocytosis, 1-iodine induced thyrotoxicosis and 1-urgent need for operation). All patients underwent plasmapheresis prior to surgery (10 patients underwent total thyroidectomy and 1 patient underwent an open reduction and internal fixation for femur fracture). The majority of patients required 1 to 3 plasmapheresis sessions to see clinical improvement and reductions in free T4 levels. Only one patient experienced a complication from plasmapheresis, a non-life threatening allergic reaction. Another patient had intraoperative bleeding from operative sites assumed to be secondary to the coagulopathy that can develop from TPE. Additionally, 50% of patients who underwent total thyroidectomy developed transient postoperative hypocalcemia. Hypocalcemia was presumed to be the result of long-term exposure to thyroid hormones with severe untreated hyperthyroidism, though postoperative hypocalcemia may have been due to parathyroid injury during surgery. Hyperthyroidism increases bone turnover which over time can lead to thyrotoxic osteodystrophy. After total thyroidectomy for hyperthyroidism, hypocalcemia can result from rapid recalcification of bones after loss of thyroid hormone stimulation [16].

In the largest retrospective study to date, 46 patients with thyrotoxicosis who were either unable to use anti-thyroid medications due to side effects, did not improve with standard thyrotoxicosis treatment or needed rapid improvement in thyroid function due to thyroid storm were treated with TPE [17]. Plasmapheresis was performed daily with sessions lasting 2.5–3 hours. The replacement fluid was FFP and the volume of replacement fluid was 1–1.5 times the calculated total plasma volume of the patient. The underlying etiology for the majority of the patients' thyrotoxicosis was Graves' disease with smaller numbers having amiodarone induced thyrotoxicosis or toxic nodular goiter. A significant improvement in free T4 levels from before to after plasmapheresis (free T4 2.9 ng/dL vs. 1.6 ng/dL, *P* ≤ 0.001) was found in all patients. When stratified by hyperthyroid diagnosis, only the patients with Graves' disease had a statistically significant reduction in free T4 levels with TPE (free T4 2.9 ng/dL vs. 1.6 ng/dL, *P* ≤ 0.001). While not statistically significant, possibly due to smaller numbers of subjects, the median free T4 did decrease after TPE in the patients with non-Graves' hyperthyroidism (free T4 3.1 ng/dL vs. 1.8 ng/dL, *P* = NS). In this study, complications related to TPE comprised of a deep vein thrombosis in a pregnant female and 2 catheter infections.

## 4. Conclusion

While current recommendations regarding TPE for treatment of thyroid storm recommend individualized decision making, little clarification regarding who would be a good candidate is available. In our case report, we present a patient with thyroid storm and multiorgan failure who was ineffectively treated with maximal medical anti-thyroid therapy alone, but was rapidly and successfully treated once plasmapheresis was added to medical anti-thyroid therapy. Our case is one of the first to show that a patient with severe critical multiorgan failure can benefit from TPE. Given our patient's extraordinary recovery and the remarkable improvements in other patients with thyroid storm treated with TPE, endocrinologists should have a lower threshold to pursue treatment of thyroid storm with plasmapheresis if patients are not improving with medical antithyroid therapy alone. Future research is needed to answer questions regarding optimal replacement fluid and volume, frequency, and duration at which TPE should proceed for thyroid storm.

## Figures and Tables

**Table 1:** Admission laboratory studies.

Lab	Result	Reference range
WBC	29.7	4.5–11.0 K/*µ*L
Hemoglobin	10.3	12.6–17.4 g/dL
Platelets	149	153–367 K/*µ*L
Creatinine	1.43	0.66–1.25 mg/dL
AST	177	17–59 units/L
ALT	44	21–72 units/L
Total bilirubin	3.8	0.3–1.2 mg/dL
Lactate	2.8	0.5–1.6 mmol/L
Troponin	7.05	≤0.06 ng/mL

## Abbreviations

MMI: Methimazole
PTU: Propylthiouracil
SSKI: Saturated potassium iodide solution
TPE: Therapeutic plasma exchange
T4: Thyroxine
TBG: Thyroxine binding globulin
T3: Triiodothyronine.
